# Two-Year Follow-Up of a Randomized Clinical Trial of Inpatient Multimodal Occupational Rehabilitation Vs Outpatient Acceptance and Commitment Therapy for Sick Listed Workers with Musculoskeletal or Common Mental Disorders

**DOI:** 10.1007/s10926-021-09969-4

**Published:** 2021-03-25

**Authors:** Lene Aasdahl, Ottar Vasseljen, Sigmund Østgård Gismervik, Roar Johnsen, Marius Steiro Fimland

**Affiliations:** 1 Department of Public Health and Nursing, Faculty of Medicine and Health Sciences, Norwegian University of Science and Technology, NTNU, Postboks 8905, 7491 Trondheim, Norway; 2 Unicare Helsefort Rehabilitation Centre, Rissa, Norway; 3 Department of Physical Medicine and Rehabilitation, St. Olavs Hospital, Trondheim University Hospital, Trondheim, Norway; 4 Department of Neuromedicine and Movement Science, Faculty of Medicine and Health Sciences, Norwegian University of Science and Technology, Trondheim, Norway

**Keywords:** Return to work, Sick leave, Musculoskeletal diseases, Mental health, Cognitive therapy

## Abstract

*Purpose* There is a lack of results on long-term effects of return to work interventions. We previously reported that an inpatient multimodal occupational rehabilitation program (I-MORE) was more effective in reducing sickness absence and facilitating return to work (RTW) at 12 months follow-up compared to an outpatient program that consisted mainly of Acceptance and Commitment Therapy (O-ACT). We now report the 2-year outcome data. *Methods* A randomized clinical trial with parallel groups. Participants were 18–60 years old, sick listed with musculoskeletal, common mental or general/unspecified disorders. I-MORE lasted 3.5 weeks and consisted of ACT, physical training and work-related problem solving. O-ACT consisted mainly of 6 weekly sessions (2.5 h. each) of ACT in groups. Outcomes were cumulated number of days on medical benefits and time until sustainable RTW (1 month without medical benefits) during 2-years of follow-up, measured by registry data. *Results* For the 166 randomized participants, the median number of days on medical benefits was 159 (IQR 59–342) for I-MORE vs 249 days (IQR 103–379; Mann–Whitney U test, p = 0.07), for O-ACT. At 2 years, 40% in I-MORE received long-term benefits (work assessment allowance) vs 51% in O-ACT. The crude hazard ratio (HR) for sustainable RTW was 1.59 (95% CI 1.04–2.42, p = 0.03) and the adjusted HR 1.77 (95% CI 1.14–2.75, p = 0.01), in favor of I-MORE. *Conclusions* The 2-year outcomes show that I-MORE had long-term positive effects on increasing work participation for individuals sick listed with musculoskeletal and mental disorders. Further follow-up and economic evaluations should be performed.

**Supplementary Information:**

The online version contains supplementary material available at 10.1007/s10926-021-09969-4.

## Introduction

Sickness absence has vast consequences at the individual and societal levels. Considerable resources are spent on preventing long-term work disability [1–3]. However, despite considerable research on the effects of return to work (RTW) interventions during the last decades, results have been inconsistent [4–7]. In a recent Cochrane review of 14 randomized controlled trials of return-to-work programs, there were no effects on RTW regardless of length of follow-up (6 months to longer than 12 months) [8]. In another systematic review, Cullen et al. [9] concluded that multimodal interventions were effective on sickness absence for individuals with musculoskeletal or mental health conditions. However, there was only a small number of high-quality studies and few with long-term follow-up. In RTW research, it is insufficient to document short term effects, as recurrent sickness absence spells are relatively common, and any effects on RTW must be sustainable to legitimate complex interventions.

In a previous paper, we reported the results at 12 months follow-up of a randomized controlled trial evaluating the effect of 3.5 weeks inpatient multimodal rehabilitation (I-MORE) for individuals sick listed due to musculoskeletal complaints or common mental disorders [10]. The program consisted of Acceptance and Commitment Therapy (ACT) [11], physical training and work-related problem solving. I-MORE was compared to a less comprehensive outpatient program, consisting mainly of ACT (O-ACT). ACT, a form of cognitive behavioral therapy, differs from traditional cognitive behavioral therapy by changing the relation to the thoughts instead of their content [11, 12]. Both negative and positive experiences should be accepted, while the person’s values should guide their actions towards their goals [11]. The aim is to increase psychological flexibility through mindfulness techniques, values and committed action [11, 13]. Effects of ACT have been shown on the main causes of sickness absence, namely chronic pain [14], anxiety [15] and depression [15, 16].

I-MORE was substantially more effective than O-ACT in facilitating sustainable RTW and reducing sickness absence days during 12 months of follow-up [10]. The length of I-MORE is in line with traditional inpatient occupational rehabilitation programs in Norway. To justify these resource intensive rehabilitation programs, the effects should be sustainable beyond 1 year. To test the sustainability of the primary findings, we now report 2-year outcome data.

## Methods

### Study Design and Participants

We conducted a randomized clinical trial with parallel groups. The trial compared I-MORE to the less comprehensive O-ACT for individuals on sick leave due to musculoskeletal or common mental disorders. The primary outcome was sickness absence during 12 months of follow-up [10]. The study protocol and several other studies have been published from this project, and the description of the methods are partly overlapping with previous studies [10, 17–21]. The study was approved by the Regional Committee for Medical and Health Research Ethics in Central Norway (No.: 2012/1241) and is registered in clinicaltrials.gov (No.: NCT01926574). The results are presented according to the CONSORT statement [22].

Eligible participants were 18 to 60 years of age who at inclusion had been sick listed 2 to 12 months with a diagnosis within the musculoskeletal (L), psychological (P) or general and unspecified (A) chapters of the ICPC-2 (International Classification of Primary Care, Second edition). Sick leave status had to be at least 50% off work at inclusion. Exclusion criteria, assessed by a comprehensive questionnaire and an outpatient screening performed by a physician, a physiotherapist and a psychologist, were: (1) alcohol or drug abuse; (2) serious somatic (e.g. cancer, unstable heart disease) or psychological disorders (e.g. high suicidal risk, psychosis, ongoing manic episode); (3) specific disorders requiring specialized treatment; (4) pregnancy; (5) currently participating in another treatment or rehabilitation program; (6) insufficient oral or written Norwegian language skills to participate in group sessions and fill out questionnaires; (7) scheduled for surgery within the next 6 months; and (8) serious problems with functioning in a group setting, as assessed by the multidisciplinary clinical team.

### The Rehabilitation Programs

*I-MORE* consisted of several components; group-based ACT, a form of cognitive behavioral therapy, individual and group-based physical training, mindfulness, education on various topics, and individual meetings with the coordinators in work-related problem-solving sessions including creating a RTW-plan. Table 1 shows an overview of the content of the program. A more detailed description can be found in the study protocol article [17]. A certified ACT-instructor supervised the coordinators who mentored the participants, both before and on a monthly basis during the intervention. ACT was chosen as the cognitive behavioral therapy approach in this study because of its applicability across diagnostic groups [12]. The program lasted 3.5 weeks with 6–7 h each day except on weekends. It took place at Hysnes rehabilitation center, which was established as a part of St. Olavs Hospital in central Norway.

**Table 1:** Overview of the two interventions

Content	
I-MORE	O-ACT
3.5 weeks inpatient program	6–7 weeks outpatient program
Acceptance and commitment therapy (group sessions; 16 h)	Acceptance and commitment therapy (group sessions; 15 h)
Physical activity (group sessions and individual guidance; total 12 h)	Discussion and advice on physical activity (group-based; 1 h)
Work-related problem solving (individual; 5 h)	Sessions with social worker (individual; 2 h)
Meeting with physician (individual; 0.5 h)	Session with social worker and ACT group moderator (individual; 0.5 h)
Mindfulness sessions (group-based; 3.5 h)^a^	Short mindfulness sessions (group; total 1.5 h) Home practice (incl. daily mindfulness)^a^
Outdoor activities day (5 h)	
“Walking to work” (3 h)^a^	
“Network day” (4 h)	
Lectures (stress, sleep, nutrition, pain; 6.5 h)	
Individual return to work plan; resume sent to GP	A short resume to the GP

*GP* general practitioner, *I-MORE* inpatient multimodal rehabilitation, *O-ACT* outpatient acceptance and commitment therapy

^a^Scheduled but not a supervised part of the program

*O-ACT* consisted mainly of group-based ACT once a week for 6 weeks, each session lasting 2.5 h. The sessions were held at the Department of Physical Medicine and Rehabilitation, St. Olavs Hospital, and was led by one of two physicians or a psychologist, all supervised by the same ACT-instructor as in I-MORE. The participants were given home assignments between sessions, including a daily 15 min audio-guided mindfulness practice. In addition, the participants were offered two individual sessions with a social worker experienced in occupational rehabilitation and trained in ACT to clarify personal values and work-related issues. The program also included a motivational group discussion with a physiotherapist on the benefits of physical training. One individual session with both the social worker and group leader present ended the program. In this session, a summary letter was written to the participant’s general practitioner. A more detailed description of the programs has been published elsewhere [10, 17].

### Study Context

All legal residents in Norway are included in the Norwegian public insurance system. Medically certified sick leave is compensated with 100% coverage for the first 12 months, with some limitations regarding the size of the salary. The first 16 days are covered by the employer, the rest by the Norwegian Welfare and Labour Administration. After 12 months of sick leave, it is possible to apply for the more long-term medical benefits, work assessment allowance and disability pension, which both covers approximately 66% of the income. Individuals on work assessment allowance are supposed to work according to their work capacity.

### Outcome Measures

Sick leave data were obtained from the Norwegian National Social Security System Registry, where all individuals receiving any form of sickness or disability benefits in Norway are registered by their social security number. Based on information from the different medical benefits (sick-leave payments, work assessment allowance and disability pension) we calculated the equivalent of full workdays on medical benefits according to a 5-day workweek for every month during follow-up [19].

Two work participation parameters were calculated: (1) cumulated number of workdays on medical benefits from inclusion to 2-years of follow-up, and (2) time until full sustainable RTW defined as 1 month without sick leave relapse, i.e. first full month without medical benefits (disregarding any graded disability the participant had when entering the study).

Other variables registered by questionnaires at inclusion were anxiety and depression symptoms measured by The Hospital Anxiety and Depression scale (HADS) [23], pain measured by one question from the Brief Pain Inventory (BPI) [24] and level of education, dichotomized as high (college/university) or low.

### Randomization and Blinding

**Fig. 1:**
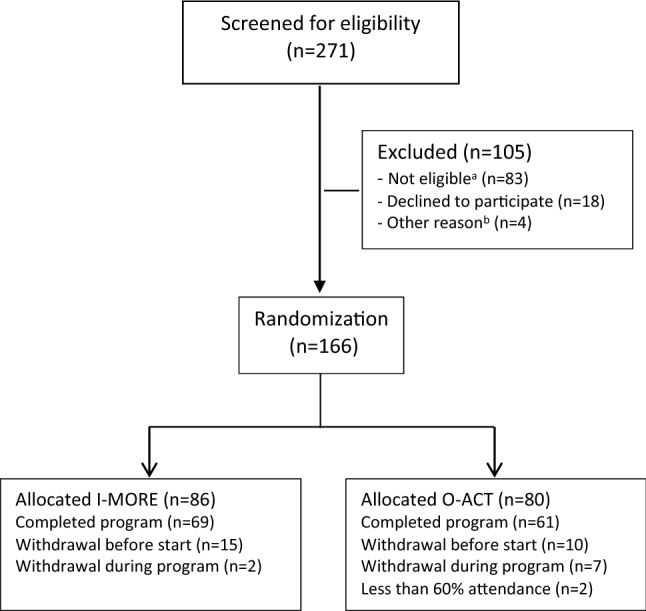
Flow of participants in the study. ^a^Not eligible: participating in another treatment program (n = 22), serious somatic/psychiatric illness (n = 11), specialized treatment needs (n = 4), problems with functioning in groups (n = 3), surgery scheduled next 6 months (n = 2), insufficient language skills (n = 2), alcohol/drug abuse (n = 1), no longer on sick-leave (n = 10), medical assessment not completed (n = 15), not motivated (n = 6), inability to participate in an inpatient intervention (n = 7). ^b^Other reason: unknown (n = 4). *I-MORE* inpatient multimodal occupational rehabilitation, *O-ACT* outpatient acceptance and commitment therapy

Potential participants were identified in the National Social Security System, between October 2012 and November 2014, and invited through a letter. Invited participants completed a short eligibility questionnaire. Those eligible were invited for the outpatient screening assessment. If the screening was passed (Fig. 1), participants were randomized to either I-MORE or O-ACT. A flexibly weighted randomization procedure was provided by the Unit of Applied Clinical Research (third-party) at the Norwegian University of Science and Technology (NTNU) to ensure that the rehabilitation center had enough participants to run monthly groups in periods of low recruitment. As a third party performed the procedure, the randomization was concealed for the researchers and participants.

It was not possible to blind neither the participants nor the caregivers for treatment. Sickness absence data was provided by employees at the Norwegian Welfare and Labor Service, who were unaware of group allocation. The researchers were not blinded.

### Statistical Analysis

Sample size was calculated based on the primary outcome, i.e. number of sickness absence days during 12 months of follow-up [10], resulting in 80 persons in each arm. Details about the estimations are published elsewhere [17].

Number of days on medical benefits from inclusion to 2-years of follow-up were calculated and compared using the Mann–Whitney U (Wilcoxon rank sum) test, as sick leave days were not normally distributed. Number of participants in the two groups who received more long-term benefits (work assessment allowance and disability pension) were compared using Pearson χ^2^ test and the Suissa-Shuster test [25, 26] for expected cell counts over and under 5 respectively. Logistic regression was used to calculate the odds ratio for receiving work assessment allowance at 2-years of follow-up. We performed the analyses both without adjustments and adjusted for gender, age, level of education, main diagnosis for sick leave and length of sick leave at inclusion. Education was dichotomized as high (university college or university) or low. For time until sustainable RTW, Kaplan Meier curves were estimated and compared with the log rank test. We estimated hazard ratios for RTW using Cox proportional hazard model with the Efron method for ties [27]. Time was calculated as number of months and participants were censored at “full sustainable RTW” or end of follow-up. We performed analyses both without adjustment and with adjustment for gender, age, level of education, main diagnosis for sick leave and length of sick leave at inclusion. The proportionality hazard assumption was checked using the Schoenfeld Residual test [28]. All analyses were performed after the intention-to-treat principle, i.e. participants were included in the analyses by their group assignment at randomization.

p-values (two-tailed) < 0.05 were considered statistically significant. Estimate precisions were assessed by 95% confidence intervals. All analyses were done using STATA 14.1 (StataCorp. 2015. Stata Statistical Software: Release 14. College Station, TX: StataCorp LP).

## Results

The flow of participants through the study is illustrated in Fig. 1. After screening, 166 individuals remained and were randomized to I-MORE (n = 86) or O-ACT (n = 80). The baseline characteristics for the participants in the two programs were comparable (Table 2).

**Table 2:** Baseline characteristics for participants

	I-MORE (n = 86)	O-ACT (n = 80)
Age mean (SD)	46.3 (8.7)	45.2 (10.4)
Women n (%)	70 (81%)	61 (76%)
Higher education^a^ n (%)	32 (37%)	34 (43%)
Work status n (%)		
No work	11 (13%)	6 (8%)
Full time	54 (63%)	53 (66%)
Part time	12 (14%)	18 (23%)
Graded disability pension	9 (10%)	3 (4%)
Sick-leave status^b^ n (%)		
Full sick-leave	35 (41%)	37 (46%)
Partial sick-leave	48 (56%)	37 (46%)
Work assessment allowance	3 (3%)	6 (8%)
Main diagnoses for sick-leave (ICPC-2)^b^ n (%)		
A- general and unspecified	5 (6%)	9 (11%)
L- musculoskeletal	54 (63%)	40 (50%)
P- psychological	27 (31%)	31 (39%)
Length of sick leave at inclusion^b,c^ median days (IQR)	204 (163–265)	216 (177–265)
Pain level, mean (SD)	5.0 (2.0)	4.8 (2.2)
HADS mean (SD)^2^		
Anxiety (0–21)	7.4 (3.9)	8.6 (4.1)
Depression (0–21)	5.7 (4.2)	6.6 (4.0)

*I-MORE* inpatient multimodal occupational rehabilitation, *O-ACT* outpatient acceptance and commitment therapy

There are some small differences from previous studies due to corrections and updated registry data

^a^Higher (tertiary) education (College or university)

^b^Based on data in the medical certificate from the National Social Security System Registry

^c^Number of days on sick leave during the last 12 months prior to inclusion. Measured as calendar days, not adjusted for graded sick- leave or part time job

### Days on Medical Benefits and Transition to Long-Term Benefits

**Fig. 2:**
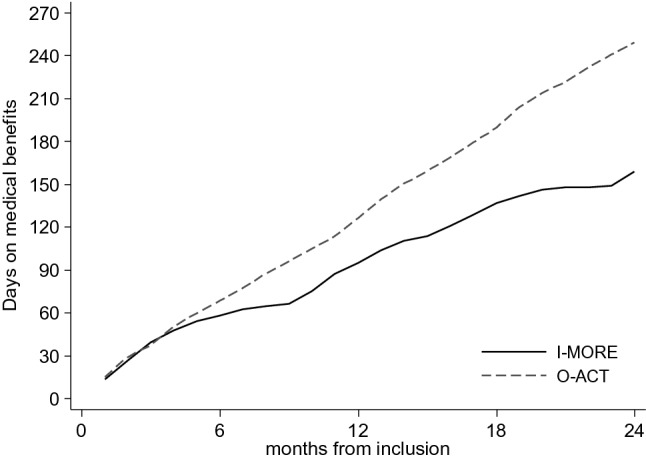
Cumulative number of work days (median) on medical benefits for the inpatient (I-MORE)- and the outpatient program (O-ACT) during 24 months of follow-up. Adjusted for employment fraction and transformed to whole workdays according to a 5-day workweek

The median number of days on medical benefits during the 2-years of follow-up was 159 (IQR 59–342) for I-MORE and 249 days (IQR 103–379) for O-ACT (Fig. 2). The between-group difference of 90 workdays did not reach statistical significance (Mann–Whitney U test, p = 0.07). During follow-up, 45 participants (54%) in I-MORE and 55 (69%) in O-ACT transitioned to work assessment allowance (p = 0.03). At 2 years, 11 (13%) of the participants in I-MORE and 5 (6%) in O-ACT received temporary sickness benefits (p = 0.15). As the time limit for such benefits is maximum 1 year, these participants could have returned to work and had a recurrent sickness absence episode, or for various reasons have been granted an extension. In total, 75 participants received work assessment allowance at 2-years of follow-up: 34 (40%) in I-MORE and 41 (51%) in O-ACT (p = 0.13). I-MORE participants were nearly half as likely to be on work assessment allowance at 2-years follow-up as O-ACT (crude OR 0.62, 95% CI 0.34–1.15, p = 0.13; adjusted OR 0.55, 95% CI 0.28–1.10, p = 0.08). At 2 years, a few participants had also transitioned to or increased their permanent disability benefit; 3 (3%) in I-MORE and 7 (9%) in O-ACT (p = 0.18).

### Sustainable RTW

**Fig. 3:**
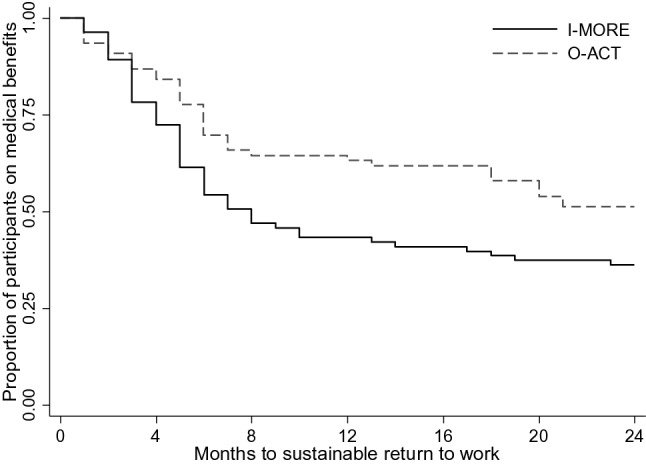
Survival curves from the Kaplan Meier analysis showing time to sustainable return to work (i.e. 1 month not receiving medical benefits) for the inpatient (I-MORE) and the outpatient (O-ACT) program

In total, 65% of the participants in I-MORE and 51% in O-ACT achieved sustainable RTW during 2-years of follow-up. Figure 3 shows the Kaplan–Meier plot, the difference between the programs was statistically significant (log rank test: p = 0.03). The unadjusted hazard ratio for sustainable RTW was 1.59 (95% CI 1.04–2.42, p = 0.03), in favor of I-MORE. The adjusted hazard ratio (adjusted for age, gender, education, main diagnosis for sick leave and length of sick leave at inclusion) was 1.77 (95% CI 1.14–2.75, p = 0.01), also in favor of I-MORE.

## Discussion

During 2-year follow-up participants in the 3.5-week I-MORE program had fewer days on medical benefits and achieved sustainable RTW faster and to a higher degree than participants in O-ACT. The results show that the outcomes from 12-month follow-up [10] are maintained at 2 years. The difference in median number of sickness absence days at 2-year follow-up was considerable (90 days), although the statistically significant difference at 12 months was not sustained. Overall, when the results from the analyses on time to sustainable RTW and the differences in transition to long-term benefits are considered, the results suggest clear benefits for I-MORE versus O-ACT.

Few previous studies have evaluated long-term sustainability of RTW interventions for individuals on long-term sick leave. Schene et al. [29] found an effect of adding occupational therapy to psychiatric treatment for individuals sick listed with major depression at 18 months of follow-up. Furthermore, a few studies found positive long-term effects of RTW interventions for individuals with shorter sick leave spells [29–32].

The RTW rates in our study were quite low for 2 years of follow-up. Jensen et al. [33] found RTW rates of 77–80% when they compared multidisciplinary intervention to brief intervention at 2-years follow-up. However, participants in that study had shorter sickness absence than in our study. In a study by Lambeek et al. [4], the participants with low back pain had been sick listed quite long, albeit not as long as in our study. They found considerably higher RTW rates already at 12 months of follow-up. The intervention in the Lambeek-study included both a workplace intervention and stakeholder coordination, which both are considered important in RTW interventions [4, 34, 35]. Nevertheless, our study suggests RTW interventions can also be effective without those components. In a previous study we found no benefit from adding a limited workplace intervention to I-MORE [36]. However, we cannot dismiss possibly larger effects if more comprehensive workplace and stakeholder involvement had been included in our study. It should be noted that the I-MORE program was quite extensive and hence costly, and an economic evaluation is necessary to decide whether the program is cost effective. A larger proportion of the participants in O-ACT transitioned to long-term benefits compared to I-MORE. If the RTW differences between the programs are maintained or increased into a difference in permanent disability benefits, it could have considerable economic consequences.

There are many possible explanations for I-MORE`s superiority. The most notable difference between the programs was the setting. Staying at the rehabilitation center for 3.5 weeks gave the participants a break from their daily life and an opportunity to focus on their own process; time for contemplation, interactions with peers, discussions and integration of new coping strategies, and physical exercise. Where the participants in I-MORE created a comprehensive RTW plan, participants in O-ACT only worked-out an action plan in accordance with their values. However, the study design precludes identifying the effective components of I-MORE. The favoring results of I-MORE could be due to single components or an interaction of several components.

Several participants withdrew before or during the study. For I-MORE, drop-outs occurred before the program started, while for O-ACT it occurred both before and during the program. For I-MORE, patients would need to pack and travel to stay at the rehabilitation center, making it a bit harder to leave when you had arrived. Whereas for O-ACT, it was possible to just not show up for the latter half of the sessions. How this might affect the results, is not straightforward to interpret. Those not starting I-MORE could be those with more problems, like social anxiety/depression, or conversely, individuals feeling very close to RTW and thus not bothering with spending 3.5 weeks on rehabilitation.

The main strength of this randomized study was the use of registry data to assess sickness absence. This ensured no recall bias or missing data. As participants were invited from the National Social Security System, there was also no referral bias. However, only about 8% accepted the invitation, which limits the generalizability of the results. Furthermore, differences in legislation and social security systems limits the generalizability of the results outside the Nordic countries. Another limitation is that the researchers were not blinded, but sickness absence was registered and provided by employees at the Norwegian Welfare and Labor Service who were unaware of group allocation. Furthermore, there was no usual care control group, meaning it is not possible to know whether the participants returned to work faster or slower than they would have without any intervention. Some minor deviations from the ClinicalTrials.gov registration (NCT01926574) should be mentioned. Follow-up data were originally planned for three and 5 years of follow-up. However, due to the natural crossroads in the Norwegian medical benefits this was changed to 2 and 7 years in order to capture the transitions to the more long-term benefits: work assessment allowance (after 12 months of sick leave) and permanent disability benefits (on average 5 to 6 years after start of sick leave period). The original planned double primary outcome was also changed from including both number of sickness absence days and time to sustainable RTW to only include the first, while the latter was changed to a secondary outcome. This was specified before the analyses were performed and published in the protocol paper [17]. Furthermore, this study was part of a larger trial including two randomized controlled trials. The sample size proposed in the ClinicalTrials.gov registration included both trials and is therefore not very precise. The more detailed sample size calculations are described in the published protocol describing the two randomized trials [17].

In conclusion, there was considerably less sickness absence (90 days) and higher RTW during the 2 years after participation in a 3.5-week I-MORE program compared to less comprehensive O-ACT for individuals sick listed due to musculoskeletal or common mental complaints. Participants in I-MORE were also less likely to transition to longer-term benefits, indicating a preventive effect on transition from sick leave to permanent work exclusion. Extended follow-up is warranted, as well as economic evaluations of the programs.

## Supplementary Information

Below is the link to the electronic supplementary material.

Supplementary file1 (DOC 230 kb)

## Data Availability

Data is not available due to ethical approval.

## Abbreviations

ACT: Acceptance and commitment therapy
HADS: The Hospital Anxiety and Depression scale
I-MORE: Inpatient multimodal occupational rehabilitation program
IQR: Interquartile range
O-ACT: Outpatient acceptance and commitment therapy
RTW: Return to work

## References

[1] OECD (2010). Sickness, disability and work: breaking the barriers.

[2] Waddell G, Burton AK (2006). Is work good for your health and well-being?.

[3] OECD (2013). Mental Health and Work: Norway. Mental Health and Work.

[4] Lambeek LC, van Mechelen W, Knol DL, Loisel P, Anema JR (2010). Randomised controlled trial of integrated care to reduce disability from chronic low back pain in working and private life. BMJ.

[5] Jensen C, Jensen OK, Christiansen DH, Nielsen CV (2011). One-year follow-up in employees sick-listed because of low back pain: randomized clinical trial comparing multidisciplinary and brief intervention. Spine.

[6] Reme SE, Grasdal AL, Lovvik C, Lie SA, Overland S (2015). Work-focused cognitive-behavioural therapy and individual job support to increase work participation in common mental disorders: a randomised controlled multicentre trial. Occup Environ Med.

[7] Finnes A, Ghaderi A, Dahl J, Nager A, Enebrink P (2017). Randomized controlled trial of acceptance and commitment therapy and a workplace intervention for sickness absence due to mental disorders. J Occup Health Psychol.

[8] Vogel N, Schandelmaier S, Zumbrunn T, Ebrahim S, de Boer WE, Busse JW (2017). Return-to-work coordination programmes for improving return to work in workers on sick leave. Cochrane Database Syst Rev.

[9] Cullen KL, Irvin E, Collie A, Clay F, Gensby U, Jennings PA (2018). Effectiveness of workplace interventions in return-to-work for musculoskeletal, pain-related and mental health conditions: an update of the evidence and messages for practitioners. J Occup Rehabil.

[10] Gismervik SO, Aasdahl L, Vasseljen O, Fors EA, Rise MB, Johnsen R (2020). Inpatient multimodal occupational rehabilitation reduces sickness absence among individuals with musculoskeletal and common mental health disorders: a randomized clinical trial. Scand J Work Environ Health.

[11] Hayes SC, Strosahl K, Wilson KG (1999). Acceptance and commitment therapy: an experiential approach to behavior change.

[12] Hayes SC, Strosahl K, Wilson KG (2012). Acceptance and commitment therapy: the process and practice of mindful change.

[13] Hayes SC, Villatte M, Levin M, Hildebrandt M (2011). Open, aware, and active: contextual approaches as an emerging trend in the behavioral and cognitive therapies. Annu Rev Clin Psychol.

[14] Wetherell JL, Afari N, Rutledge T, Sorrell JT, Stoddard JA, Petkus AJ (2011). A randomized, controlled trial of acceptance and commitment therapy and cognitive-behavioral therapy for chronic pain. Pain.

[15] Forman EM, Herbert JD, Moitra E, Yeomans PD, Geller PA (2007). A randomized controlled effectiveness trial of acceptance and commitment therapy and cognitive therapy for anxiety and depression. Behav Modif.

[16] Folke F, Parling T, Melin L (2012). Acceptance and commitment therapy for depression: a preliminary randomized clinical trial for unemployed on long-term sick leave. Cogn Behav Pract.

[17] Fimland MS, Vasseljen O, Gismervik S, Rise MB, Halsteinli V, Jacobsen HB (2014). Occupational rehabilitation programs for musculoskeletal pain and common mental health disorders: study protocol of a randomized controlled trial. BMC Public Health.

[18] Aasdahl L, Gismervik SO, Marchand GH, Vasseljen O, Johnsen R, Fimland MS (2019). Changes in fear-avoidance beliefs and work participation after occupational rehabilitation for musculoskeletal- and common mental disorders: secondary outcomes of two randomized clinical trials. J Rehabil Med.

[19] Aasdahl L, Pape K, Vasseljen O, Johnsen R, Gismervik S, Halsteinli V (2017). Effect of inpatient multicomponent occupational rehabilitation versus less comprehensive outpatient rehabilitation on sickness absence in persons with musculoskeletal- or mental health disorders: a randomized clinical trial. J Occup Rehabil.

[20] Aasdahl L, Pape K, Vasseljen O, Johnsen R, Gismervik S, Jensen C (2016). Effects of inpatient multicomponent occupational rehabilitation versus less comprehensive outpatient rehabilitation on somatic and mental health: secondary outcomes of a randomized clinical trial. J Occup Rehabil.

[21] Aasdahl L, Pape K, Vasseljen O, Johnsen R, Fimland MS (2018). Improved expectations about length of sick leave during occupational rehabilitation is associated with increased work participation. J Occup Rehabil.

[22] Schulz KF, Altman DG, Moher D (2010). CONSORT 2010 Statement: updated guidelines for reporting parallel group randomised trials. BMC Med.

[23] Zigmond AS, Snaith RP (1983). The hospital anxiety and depression scale. Acta Psychiatr Scand.

[24] Cleeland CS, Ryan KM (1994). Pain assessment: global use of the brief pain inventory. Ann Acad Med Singapore.

[25] Lydersen S, Langaas M, Bakke Ø (2012). The exact unconditional z-pooled test for equality of two binomial probabilities: optimal choice of the Berger and Boos confidence coefficient. J Stat Comput Simul.

[26] Suissa S, Shuster JJ (1985). Exact unconditional sample sizes for the 2 times 2 binomial trial. J R Stat Soc A (General).

[27] Efron B (1977). The efficiency of Cox's likelihood function for censored data. J Am Stat Assoc.

[28] Schoenfeld D (1982). Partial residuals for the proportional hazards regression model. Biometrika.

[29] Schene AH, Koeter MW, Kikkert MJ, Swinkels JA, McCrone P (2007). Adjuvant occupational therapy for work-related major depression works: randomized trial including economic evaluation. Psychol Med.

[30] Lindstrom I, Ohlund C, Eek C, Wallin L, Peterson LE, Fordyce WE (1992). The effect of graded activity on patients with subacute low back pain: a randomized prospective clinical study with an operant-conditioning behavioral approach. Phys Ther.

[31] Karjalainen K, Malmivaara A, Mutanen P, Roine R, Hurri H, Pohjolainen T (2004). Mini-intervention for subacute low back pain: two-year follow-up and modifiers of effectiveness. Spine.

[32] Indahl A, Haldorsen EH, Holm S, Reikerås O, Ursin H (1998). Five-year follow-up study of a controlled clinical trial using light mobilization and an informative approach to low back pain. Spine.

[33] Jensen C, Jensen OK, Nielsen CV (2012). Sustainability of return to work in sick-listed employees with low-back pain. Two-year follow-up in a randomized clinical trial comparing multidisciplinary and brief intervention. BMC Musculoskelet Disord.

[34] Anema JR, Steenstra IA, Bongers PM, de Vet HC, Knol DL, Loisel P (2007). Multidisciplinary rehabilitation for subacute low back pain: graded activity or workplace intervention or both? A randomized controlled trial. Spine.

[35] Bultmann U, Sherson D, Olsen J, Hansen CL, Lund T, Kilsgaard J (2009). Coordinated and tailored work rehabilitation: a randomized controlled trial with economic evaluation undertaken with workers on sick leave due to musculoskeletal disorders. J Occup Rehabil.

[36] Skagseth M, Fimland MS, Rise MB, Johnsen R, Borchgrevink PC, Aasdahl L (2019). Effectiveness of adding a workplace intervention to an inpatient multimodal occupational rehabilitation program: a randomized clinical trial. Scand J Work Environ Health.

